# Thermal optima of cercarial emergence in trematodes from a marine high-temperature ecosystem, the Persian Gulf

**DOI:** 10.1038/s41598-023-31670-0

**Published:** 2023-03-25

**Authors:** Maral Khosravi, Dakeishla M. Díaz-Morales, David W. Thieltges, Martin Wahl, Jahangir Vajedsamiei

**Affiliations:** 1 Department of Marine Ecology, GEOMAR Helmholtz Centre for Ocean Research Kiel, Düsternbrooker Weg 20, 24105 Kiel, Germany; 2 Department of Aquatic Ecology and Centre for Water and Environmental Research, University of Duisburg-Essen, Universitatsstr. 5, 45141 Essen, Germany; 3 Department of Coastal Systems, NIOZ Royal Netherlands Institute for Sea Research, PO Box 59, 1790 AB Den Burg Texel, The Netherlands

**Keywords:** Climate-change ecology, Ecological epidemiology

## Abstract

Global warming may alter the dynamics of infectious diseases by affecting important steps in the transmission of pathogens and parasites. In trematode parasites, the emergence of cercarial stages from their hosts is temperature-dependent, being highest around a thermal optimum. If environmental temperatures exceed this optimum as a consequence of global warming, this may affect cercarial transmission. However, our knowledge of cercarial emergence patterns of species from high temperature environments is currently very limited. Here, we investigated the effect of temperature on the emergence of two common trematode species from an abundant mud snail *Pirenella cingulata* in the Persian Gulf, the warmest sea on Earth. Infected snails were incubated in the laboratory at 6 temperatures from 10 to 40 °C for 3 days. We found an optimal temperature for cercarial emergence of 32.0 °C and 33.5 °C for *Acanthotrema tridactyla* and Cyathocotylidae gen. sp., respectively, which are the warmest recorded thermal optima for any aquatic trematode species. Emergence of both species dropped at 40 °C, suggesting upper thermal limits to emergence. Overall, Persian Gulf trematodes may be among the most heat-tolerant marine trematode species, indicating a potential for dispersing to regions that will continue to warm in the future.

## Introduction

Global warming is expected to have profound effects on biological systems at every level of their organization, from molecules to whole ecosystems^1,2^. These effects include, but are not limited to, altering organismal functioning and life history, population dynamics, and species dispersal^3–6^. In addition to affecting free-living species, it is anticipated that global warming will alter pathogen and parasite outbreaks, particularly through effects on transmission dynamics^7–9^. Studies have demonstrated, for instance, that warming can directly boost infection levels in hosts by increasing the production of infective stages of parasites^10^, expanding seasonal infection windows^11^, elevated movement activity of cercariae thus increasing chances to find a host^12^, and intensifying the susceptibility of hosts by reducing host immune response^13–16^. Since the effects of warming on parasite transmission dynamics can have far-reaching ecological and economic consequences for hosts and ecosystems^9,17^, it is important to understand the effects of temperature on parasite transmission as a pre-requisite for predicting climate change effects on the biosphere.

Trematodes are an excellent example of parasites for which temperature effects are well known to affect crucial steps in the transmission process from one host to another^18,19^. Their life cycle usually involves three different hosts: a mollusc as first intermediate host, invertebrates or vertebrates (depending on the species) as second intermediate host, and vertebrates as final host^20^. Apart from trophic transmission to final hosts, trematodes transmit via free-living larval stages, which are at the mercy of the external environment^21^. Cercarial stages are responsible for the transmission from the first to the second intermediate host, and their emergence from the first intermediate hosts is temperature dependent^10,22^. Cercarial emergence usually occurs within a specific temperature window in which the conditions are optimal for cercariae functional activity appropriate for transmission to the downstream host^23,24^. Within this window, cercarial emergence is often very low at the lower temperature end, then increases towards an optimum, and often declines again at the higher end of the temperature window^10,24,25^.

In general, the optimal temperature ranges for cercarial emergence usually exhibit a latitudinal decline from 20 to 30 °C at low latitudes (≤ 35° of latitude) to 15–25 °C at mid-latitudes (36–60°)^22^. Potential temperature limits to cercarial emergence would be highly relevant for anticipating climate change effects on cercarial transmission dynamics. However, our knowledge of cercarial emergence patterns of species from high-temperature environments at low latitudes is very limited and largely restricted to a few freshwater trematode species of medical or veterinarian importance^22^. Likewise, little is known about how cercarial emergence in high-temperature environments interacts with other abiotic factors such as light^26^, which can sometimes lead to distinctive circadian rhythms, with some species showing higher cercarial emergence during the day and others at night ^25,27,28^.

An ideal system to conduct research on the effects of temperature on cercarial emergence in species from high-temperature environments is the Persian Gulf, the warmest sea on Earth. Due to its shallow depth and restricted water exchange with the open ocean, the sea surface temperature (SSTs) in its various locations can fall as low as 13 °C in the winter^29^ and reach to 35 °C in the summer. The frequency of heatwaves surpassing 36 °C has increased 19-fold since 1997^30^, with a recent extreme record of 37.6 °C in SSTs on 30th July 2020^31^. Thus, hosts and parasites inhabiting the intertidal zones of the Persian Gulf are eurytherms with a broad temperature tolerance window. Among the trematode hosts are gastropod species from the genus *Pirenella* which can occur in densities of up to 2100 ind.m^−2^^32^ and endure a wide range of salinity (1.2–60)^33^ and temperature (5–45 °C)^34^. *Pirenella* species serve as the first intermediate host for several trematode species^35–37^. The most abundant *Pirenella* species along the intertidal zone of the Persian Gulf is *Pirenella cingulata,* a species that is currently found to inhabit the Indo-West Pacific from India and Sri Lanka to Papua New Guinea, the northern coast of Japan and south to central Queensland (Supplementary Fig. S1). *P.* *cingulata* can be infected by 28 trematode species belonging to 10 families along the Persian Gulf coastline (Khosravi et al. unpublished data). From these, the most common trematode species are *Acanthotrema tridactyla*^38^ (Sohn et al. 2003)^39^, a cercaria with parapleurolophocercous morphology and a prevalence of up to 18%, and the morphospecies (still pending formal description) ‘Cyathocotylidae gen. sp.’, a cercaria with furcocercous morphology and a prevalence of up to 12% (Khosravi et al. unpublished data). *A. tridactyla* pertains to the family Heterophyidae, has a median dorso-ventral fin-fold on the tail, is an active swimmer, uses eye spots to locate the next host (the fish *Aphanius dispar*^40,41^, and has rediae as the intra-gastropod stage (parthenitae). Metacercariae of *A. tridactyla* have been found under the scale of laboratory-infected *Gambusia* and in the connective tissue of the head and visceral region of naturally infected *Aphanius fasciatus* in Red Sea, Egyptian intertidal coasts^38^. Final host of genus *Acanthotrema* (previously *Stictodora*) are piscivorous birds and mammals, including humans^38–44^. ‘Cyathocotylidae gen. sp.’ belongs to the Cyathocotylidae family. Although the life cycles of the members of this family remain largely unresolved, they are known to use fish, amphibians, or crustaceans as second intermediate host, and reptiles, birds or mammals, as final host^45,46^. Yet, it’s cercariae is an active swimmer, has a bifurcated tail, no eyespots, and has sporocysts as parthenitae. The thermal biology of these trematodes is unknown, and progress in this area can shed light on their future transmission dynamics in the warming Persian Gulf and surrounding regions.

This study sought to determine the temperature dependence of cercarial emergence of two trematode species (*A. tridactyla* and Cyathocotylidae gen. sp.) infecting *P. cingulata* snails in the Northern Persian Gulf. In our laboratory experiment, we measured the emergence of cercariae across a broad range of experimental temperatures which were based on sea surface temperatures measured at our sampling site (Genaveh, located at 29° 33′ 14.022′′ N, 50° 30′ 34.0416′′ E in the Northern Persian Gulf) over the last twelve years (2010–2022). The experimental temperatures ranged from 10 to 34 °C, with an additional extreme temperature scenario of 40 °C. In addition, we investigated whether these emergence patterns were affected by circadian rhythms (exposure to day and night). Our research contributes to our extremely limited knowledge of cercarial emergence in marine high-temperature environments at low latitudes.

## Results

### Cercarial emergence in response to temperature, light regime, and time

**Figure 1:**
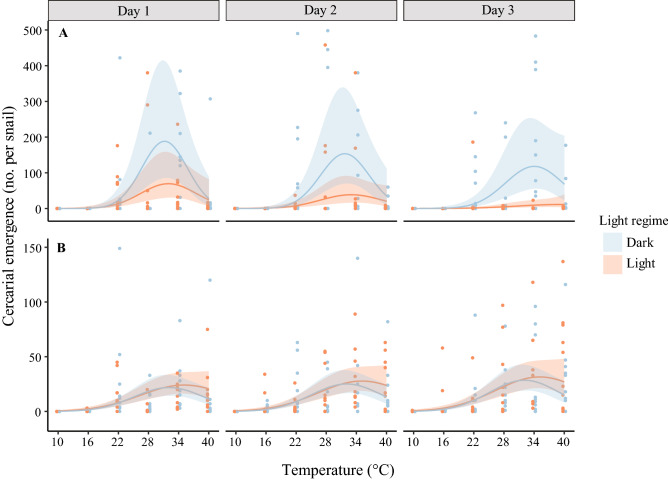
Generalized linear mixed models’ predictions for the mean (lines) and 95% confidence intervals (shaded areas) of cercarial emergence in response to *temperature*, *light regime*, and *time*, presented for two studied trematode species *Acanthotrema tridactyla* (**A**) and Cyathocotylidae gen. sp. (**B**). The number of cercariae emerged from snails was counted in 12 h intervals (*dark* and *light* periods) over 3-day experiments, represented by blue (*dark*) and orange (*light*) points.

The best-fit models adequately explained variation in cercarial emergence of *Acanthotrema tridactyla* (R^2^c = 0.82) and Cyathocotylidae gen. sp. (R^2^c = 0.61). Both species of trematode exhibited temperature-dependent (curvilinear) cercarial emergence under all experimental days and light regimes (Fig. 1). *A. tridactyla* showed a generally more abundant emergence than Cyathocotylidae gen. sp. which decreased over time and exhibited a clear circadian pattern (Fig. 1, Table 1). In contrast, Cyathocotylidae exhibited a less abundant cercarial emergence independent of light or time (Fig. 1, Table 1). The results of the Wald tests for the significance of the models’ estimated parameters representing the primary and interactive effects of *temperature*, *light regime*, and *time* on cercarial emergence are presented in Table 1.

**Table 1:** Wald test results for the significance of quasipoisson linear mixed models’ parameter estimates which are linked to main and interactive effects of *temperature*, *light regime*, and *time* on cercarial emergence, for trematode species *Acanthotrema tridactyla* (A) and Cyathocotylidae gen. sp. (B).

(A) *Acanthotrema tridactyla*	Estimate	Std. Error	z value	Pr( >|z|)
(Intercept)	1.173	0.506	2.317	0.020**
Light8	0.671	0.382	1.759	0.078
Poly (Temperature, 2)1	40.777	10.607	3.844	0.000***
Poly (Temperature, 2)2	− 20.582	8.288	− 2.483	0.013*
Poly (Day, 2)1	− 11.276	4.282	− 2.633	0.008**
Poly (Day, 2)2	− 3.965	3.767	− 1.053	0.292
Light8: Poly (Temperature, 2)1	14.870	8.486	1.752	0.079
Light8: Poly (Temperature, 2)2	− 14.106	5.969	− 2.363	0.018*
Light8: Poly (Day, 2)1	12.876	2.941	4.378	0.000***
Light8: Poly (Day, 2)2	3.761	2.734	1.376	0.169
Poly (Temperature, 2)1: Poly (Day, 2)1	− 41.871	82.977	− 0.505	0.613
Poly (Temperature, 2)2: Poly (Day, 2)1	122.798	56.55	2.171	0.029*
Poly (Temperature, 2)1: Poly (Day, 2)2	8.389	76.118	0.110	0.912
Poly (Temperature, 2)2: Poly (Day, 2)2	20.906	53.340	0.392	0.695

(B) Cyathocotylidae gen. sp	Estimate	Std. Error	z value	Pr( >|z|)
(Intercept)	1.984	0.184	10.760	0.000***
Light8	− 0.342	0.188	− 1.814	0.069
Poly (Temperature, 2)1	25.326	3.672	6.897	0.000***
Poly (Temperature, 2)2	− 10.989	3.235	− 3.397	0.000***
Poly (Day, 2)1	2.070	1.075	1.925	0.054
Poly (Day, 2)2	− 0.055	1.027	− 0.054	0.957
Light8: Poly (Temperature, 2)1	2.641	4.148	0.637	0.524
Light8: Poly (Temperature, 2)2	− 6.353	3.278	− 1.938	0.052

The significant effects have p‐value < 0.05. *light regime* had two levels: *Light20* and *Light8* representing 12-h light and dark periods, respectively. *Poly (predictor, 2)1* and *Poly (predictor, 2)2* represent linear and quadratic effects of the predictor, respectively.

Signif. codes: 0 ‘***’ 0.001 ‘**’ 0.01 ‘*’ 0.05 ‘.’ 0.1 ‘ ’ 1.

**Figure 2:**
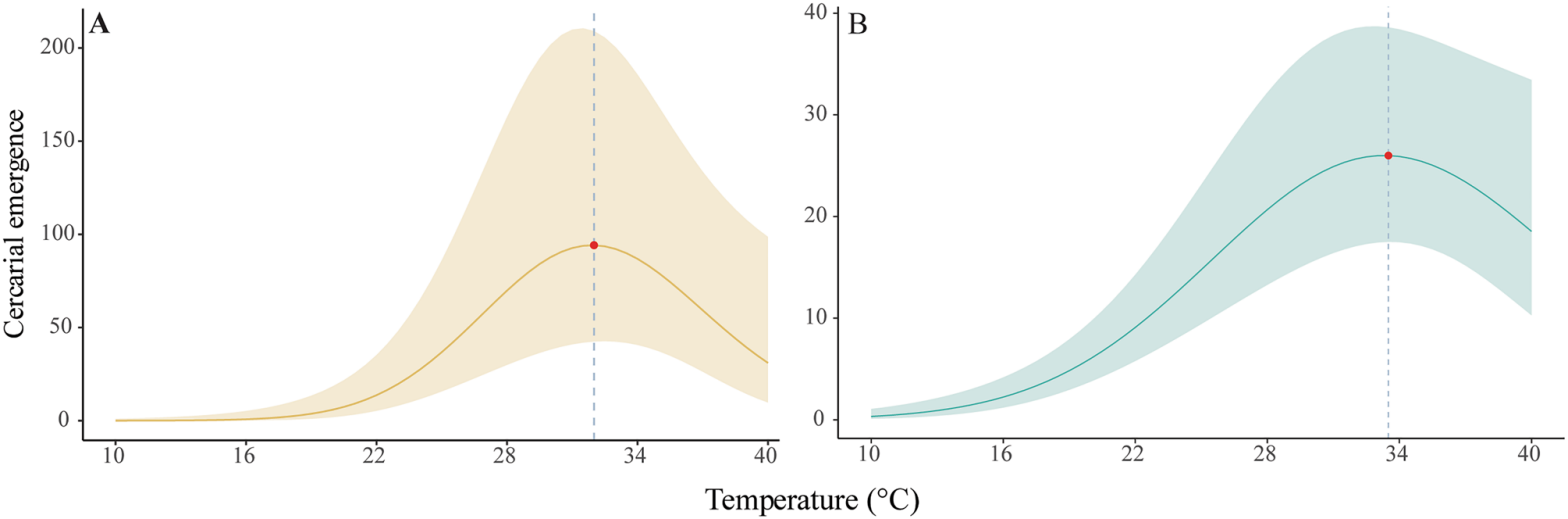
Overall effects of the temperature on cercarial emergence of *Acanthotrema tridactyla* (**A**) and Cyathocotylidae gen. sp. (**B**). Generalized linear mixed models’ predictions of the mean (lines) and 95% confidence intervals (shaded areas) were averaged for each temperature. The red dots and grey dashed lines depict cercarial emergence optimal temperatures (i.e., 32.0 °C and 33.5 °C for *A. tridactyla* and Cyathocotylidae gen. sp., respectively).

The overall effect of temperature on cercarial emergence was significant for both trematode species (Table 1; Fig. 2). *A. tridactyla* and Cyathocotylidae gen. sp. had the highest mean cercarial emergence rates of 94 and 26 cercariae per snail, respectively, at temperatures of 32.0 °C and 33.5 °C (Fig. 2). At temperatures above these optima, there was a trend for cercarial emergence to decline for both species.

Based on raw data, for both species, 10 °C was the only temperature with zero emergence, thus representing MDTT, whereas 16 °C was the METT with less than 20 emerged cercariae per snail for both parasite species. During the 3-day incubation experiment, the highest number of emerged cercariae from a single snail within 24 h was 2798 at 28 °C for *A. tridactyla* and 520 at 34 °C for Cyathocotylidae gen. sp.

For *A. tridactyla*, the non-linear effects of temperature on cercarial emergence significantly changed in response to the light regime (*p* = 0.018; Table 1). In addition, the main effect of the light regime on cercarial emergence was marginally insignificant (*p* = 0.078; Table 1) as the species tended to have increased cercarial emergence during *dark* or night time (Fig. 1). Based on the model prediction, the absolute effect size was 2.4 times higher emergence in *dark* compared to *light*. However, at the optimal temperature range (ca. 28–34 °C), the emergence was up to 2.8 times higher in *dark* than *light*. Besides, cercarial emergence significantly decreased with time (*p* = 0.008; Table 1). The interactive effect of light regime and time on emergence was also significant (*p* < 0.001; Table 1), represented by an increased influence of light regime with time (Fig. 1). The non-linear effect of temperature also significantly changed over time (*p* = 0.013; Table 1).

In the case of Cyathocotylidae gen. sp., a change in the non-linear effect of *temperature* in response to the *light regime* was found marginally insignificant (*p* = 0.052; Table 1). The main effect of *light regime* on emergence was insignificant (*p* = 0.069; Table 1), while the effect of *light regime* combined with quadratic effects of *temperature* was marginally insignificant (*p* = 0.052). A trend of increase in emergence over time was also found marginally insignificant (*p* = 0.054, Table 1). Based on the model prediction, the interaction between light and temperature was also marginally insignificant (*p* = 0.052; Table 1) (Fig. 1). The data are accessible in supplementary material.

## Discussion

Cercarial emergence from the first mollusc host is an important step in transmission, dispersion and continuation of trematode life cycles and, as a result, disease dynamics. Cercarial emergence is temperature-driven and optimized at temperatures representing thermal optima for cercariae functioning^11,47^. Considering ongoing global ocean warming, understanding the thermal sensitivity of cercarial emergence can help us predict the future transmission dynamics of trematode species. However, this knowledge is largely lacking for marine trematodes living in high-temperature environments.

### High thermal optima of cercarial emergence

Temperature facilitates the transition of parthenitae from the formation of one type of embryo to another, speeding up the embryogenesis process. This acceleration of embryogenesis involves the increase in the appearance of both young rediae and/or sporocysts of different generations that ultimately release cercariae^20^. Cercarial emergence commonly increases up to an optimum temperature above which it declines^10,22,48^. This thermal optimum usually varies due to genetic adaptation of the trematode and snail host and can depend on their acclimating (micro)habitat conditions^22,49^. Previous studies have shown that the temperature optimum for cercarial emergence decreases with latitude, from 20 to 30 °C at low latitudes (35°) to 15 to 25 °C at mid-latitudes (36–60°)^22^. The trematode species studied herein, *A. tridactyla* and Cyathocotylidae gen. sp., exhibited a maximum emergence at 32.0 °C and 33.5 °C, respectively, which are the warmest recorded thermal optima so far for any aquatic trematode species^22^. These findings suggest that both parasite species are adapted to the extreme thermal habitats in the Persian Gulf. In the sampling region (northwest Persian Gulf), the monthly average SST has varied between 28 and 36 °C, with an average of ca. 32.0 °C during the summers of 2010–2022 (Supplementary Fig. S2; Data courtesy of NOAA coast watch). Therefore, summer might be the peak season for the cercarial emergence of these two species infecting *P.* *cingulata*. Nevertheless, these intertidal habitats can also experience wide diurnal temperature ranges in summer (e.g., ~ 30–55 °C; Bordbar et al. unpublished data)^50–52^, which, in combination with tidal aerial exposures in summer, might be restrictive for cercarial emergence. Moreover, although seasonality was not directly assessed in this study, many studies suggest that seasonality plays a major role in cercarial emergence. In fact, some studies suggest that seasonality might have a stronger influence on emergence and transmission than temperature itself. For instance, de Montaudouin et al.^25^ reported seasonality in the accumulation of *H. quissetensis* in cockles at Oualidia, Morocco, where the temperature fluctuates slightly along the year. Therefore, we suggest future studies tackling the seasonality of the Persian Gulf trematodes to weigh the influence of seasonality and temperature on trematode transmission.

Information on the seasonality of trematodes from the Persian Gulf is not available, however, since closely-related species with similar hosts, transmission strategies and searching behaviours share similar emergence rhythms^53^, certain inferences can be drawn based on other described species. Trematodes of the family Heterophyidae, such as *A. tridactyla,* are known to have active rediae throughout the year, while for Cyathocotylidae gen. sp., the phenology of intramolluscan stages had not yet been described. However, for Diplostomidae, a better-studied and closely related family to Cyathocotylidae^54^, parthenita (sporocyst in this case) activity is not arrested during winter (i.e., at colder temperatures)^55^. This resembles the seasonal cercarial emergence patterns of Heterophyids. The similarity in the overwintering capacity of the larval stages of both species studied herein is supported by the minimum emergence temperature threshold (METT) values, which was ca. 16 °C (based on our raw data) for both *A. tridactyla* and Cyathocotylidae gen. sp.

Notably, the Persian Gulf’s SST has warmed up, on average, by 0.04 °C year^-1^ since the 1980s, which is double the global average^31,56^, and by the year 2100, the SST is projected to increase by another 2–4 °C, depending on the global emission scenario^30,31,57^. Despite the fact that we reported a high thermal optimum for cercarial emergence (32.0 and 33.5 °C for *A. tridactyla* and Cyathocotylidae gen. sp. respectively), our results demonstrate a drop in the emergence around 40 °C, which might indicate the onset of beyond-optimal conditions. Therefore, the seasonality of cercarial release in the Persian Gulf may change in the future, in the absence of further heat adaptation, shifting from summer to spring or fall in the Persian Gulf. Furthermore, the warming of the Indian ocean and other marine regions may provide new habitats for warm-adapted trematodes such as Persian Gulf trematodes. By now, *A. tridactyla* has been only reported from intertidal habitats in the Egyptian Red Sea, which also experiences high temperatures in summer (up to ca. 30 °C)^38^. The establishment and subsequent flourishment of trematodes outside their current range will depend on the presence of the host species needed to complete the life cycle. While knowledge about other intermediate hosts of the studied trematodes is limited, the first intermediate host *P. cingulata* of both trematode species and the second intermediate host of *A. tridactyla*, the fish *A. dispar*^40,41^, are both eurytherms with vast geographic distributions^34,58,59^ (see the Supplementary Fig. S1). Many migrating birds, which may be the trematodes’ ultimate hosts, use the Persian Gulf islands and shoreline for resting, feeding, and/or mating^60–62^, and may facilitate trematode invasions originating from the Persian Gulf. Due to the huge amount of shipping to and from this maritime area^63^, ships may also contribute to the co-invasion of trematodes along with their hosts^64^.

### Other important aspects of cercarial emergence

Successful transmission from the first to second intermediate host is a challenge in the life cycle of trematodes. Given the short life span of cercariae (< 24 h), they have adapted their emergence patterns to optimize successful rendezvous transmission to the next host^65^. In general, even at optimal temperature, cercarial emergence will not stay constant because it depends on the developmental intramolluscan stage of trematode. Cercariae of some trematodes continuously emerge from the host, while in other cases, cercariae emerge in pulses followed by a period of recovery^20^. We observed a significant decline in emerged cercariae for *A. tridactyla* over time (see Table 1 and Fig. 1), suggesting that this parasite’s redial maturation may follow a pulsed-emergence strategy, potentially followed by a regeneration period. This pulsed-emergence strategy is characteristic of trematodes having second-intermediate hosts in low abundances, and, generally, such trematodes show high synchrony with the host spatial distribution and timing of activities^20^. In contrast, our result showed a marginally insignificant increase in cercarial emergence over time for Cyathocotylidae gen. sp., which may suggest that the emergence and sporocyst maturation is rather continuous and random. For diplostomoids, such as some members of Cyathocotylidae, the second-intermediate host is usually fish^66^. Such random and continuous cercarial emergence can provoke the accumulation of metacercariae in fish, a pattern that has been observed in other diplostomoid-fish systems^67^. However, because the life cycle of members of the Cyathocotylidae family is largely unknown, this remains speculative. The different temporal emergence pattern and the higher number of emerged cercariae of *A. tridactyla* compared to Cyathocotylidae gen. sp. might be because the downstream host of the latter is more abundant than the former. Nevertheless, it is important to keep in mind that this study monitored cercarial emergence only for three days. A longer time frame (e.g., weeks-long incubations) might be better suited to evaluate the combined effect of temperature and time on the development of trematode intramolluscan stages and, ultimately, cercarial emergence.

Circadian cercarial emergence rhythms are genetically controlled^65^, and correspond with the activity of the second intermediate hosts “host-time” while avoiding contact with the regular predators^68–73^. The rhythm is often linked to rough transition between periods of light and darkness rather than minor influence of intensity or quality of light^74^. Prokofiev et al.^53^ and Uspenskaya^75^ observed a similar phenomenon in which crabs were entering the intertidal zone in the evening and presented a rather quiescent behaviour during the night, the time at which *Microphallus similis* emerged and actively searched for its second intermediate host. Some cercariae can detect light by the use of photoreceptors (eye spots) that allow them to swim towards (positive phototaxis; e.g., *Cryptocotyla lingua*) or away from the light (negative phototaxis; e.g., *Trichobilharzia szidati*)^76,77^. The results of the present study showed a clear nocturnal pattern in cercarial emergence for *A. tridactyla*. The cercaria of this species has eye spots that, as described above, work as photoreceptors for orientation and host finding. Its second intermediate host is the fish *Aphanius dispar*. *A. dispar* is a eurythermal and euryhaline fish species of coastal lagoons. The wide range of temperature and salinity tolerance enables it to also exist in freshwater river systems, hot sulfur-rich springs, and hypersaline conditions^78–80^. Individuals of *A. dispar* are active during the day and relatively quiescent during the night^78^. A higher emergence during the host’s resting time over the night might offer an advantage since the fish is conglomerated in the same place for a limited time and provide a convenient situation for the parasite by providing a point-static target for the cercariae to penetrate its skin. Therefore, it makes sense that a high number of cercariae are shed at once to maximize infection intensity in the next host. There are four hypotheses concerning how cercariae react to light changes: (1) The snail hosts act as mediators (e.g. via behavioural alterations) between the external environment and intra-molluscan larval stage of parasite; (2) The cercariae themselves are sensing and reacting to the light; (3) The daughter sporocysts (or rediae) are causing the rhythmic phenomenon; and (4) Both cercariae and daughter sporocysts are involved, separately or consecutively, with two possibilities of information transmission: from cercariae to sporocyst or vice versa^65^. Notably, our study did not describe the exact timing of cercarial release. This information will be needed to test the above hypotheses regarding the relationship between circadian rhythm of cercarial release, cercaria phototaxis, and host behaviour.

### Variability in cercarial emergence among and within trematode species

The considerable variance in cercarial production can be attributed to the sizes of both the parasites and the hosts^81^. In our study, Cyathocotylidae gen. sp. cercariae (ca. 1000 µm body length) are two times larger than the ones of *A. tridactyla* (ca. 500 µm body length; Supplementary Fig. S3). The former also had 3.6 times lower cercarial emergence than the latter at their optimal temperature. When comparing trematode species infecting the same host species of equal size (as done in this study), usually trematodes with smaller cercarial sizes may have more space in the host to produce more cercariae and thus may show higher emergence^82^. In contrast, trematodes with larger cercariae (i.e., Cyathocotylidae gen. sp.) will inherently have fewer cercariae emerging from the host. Our results are consistent with prior research that revealed a negative correlation between daily cercarial output and cercariae size for 12 trematode species in the Barents and White Seas^53^. The abundance of emerged cercariae may also depend on the release pattern and might be linked to the abundance of the downstream host^83^. In terms of cercarial production and activity, parasite genotypes may respond differently to temperature^84^. The size and physiological state of the snail host at the time of infection may affect cercarial output by determining available resources. The bigger the snail host at the moment of infection, the more developed its gonad, resources, and space. Due to variations in inherent immunity, certain snail genotypes may be easier for the parasite to exploit^81^. We randomly collected snails from the field in our investigation and chose those with patent infections (emerging cercariae). Therefore, we did not know about these factors and the age of infection; as such, its contribution to the data variance would be speculative.

## Conclusions

The Persian Gulf is the Earth’s warmest sea regarding the maximum seasonal temperature and its host-parasite systems are adapted to high temperatures, as suggested by the results of this study. The trematode species investigated here adopt highly tolerant intermediate hosts (*P. cingulata* and *A. dispar)*, which are highly warm tolerant and widely distributed, suggesting that both trematode species could expand to new habitats under ongoing global warming. Further research is needed to determine the heat sensitivity of additional trematode stages, such as cercariae survival and infection success in this extreme environment. To understand disease dynamics, this research must also incorporate cercarial infectivity and survival, the infected downstream host’s survival, and the effects of additional abiotic stressors like desiccation, hyper salinity, and eutrophication on trematode transmission.

## Materials and methods

### Host and trematode collection

On a single occasion in February 2020, several hundreds of *Pirenella cingulata* host snails were haphazardly collected by hand at low tide from the upper intertidal zone of a site known from previous research to have high trematode infection levels (Genaveh in the Northern Persian Gulf; 29°33'14.022"N, 50°30'34.0416"E; (Khosravi et al. unpublished data)). The samples were transported by flight to GEOMAR Helmholtz Center for Ocean Research Kiel, Germany, where they were kept in groups of 100 individuals in 2 L tanks for two weeks before starting the experiment. Each tank was filled with aerated reconstituted seawater (37 ± 1) (mixing Red Sea Salt^®^ and deionized water) and kept in a thermo-bath set to 18 °C with a 12:12 photoperiod (start of sunrise at 6:00, maximum light intensity at 12:00 (50% of the maximum possible intensity with irradiance ~ 445 µmol m^-2^ s^-1^, and complete sunset at 18:00). The light:dark cycles were made using Smart Reef LED (Hydra^®^ 32HD). Snails were fed ad libitum with dried powder of *Chlorella vulgaris* (Algomed^®^), and the water of each tank was changed every other day*.* In order to detect snails with patent infections (infections presenting developed cercarial emergence), the snails were placed individually in 6-well plates filled with 10 mL of aerated reconstituted seawater at 25 ± 2 °C under constant illumination for 3–5 h. Each well plate was inspected for emerged cercariae under a stereo-microscope (Nikon, SMZ1000 body, C-PS160 stand). Infected individuals (identified by emerged cercariae) were kept in separate containers based on their infection status in the same conditions explained above for 4 days before starting the experiment.

### Experimental design

A total number of 20 snails, half infected by *A. tridactyla* or Cyathocotylidae gen. sp., were assigned to each experimental target temperature (10, 16, 22, 28, 34, 40 °C). The first five temperatures represented normal to extreme SSTs observed over the past decade (Supplementary Fig. S2), and the last one represented an end-of-century heatwave temperature, given a projected 4 °C increase^85^ in the baseline temperature of a typical heatwave^30^.

The snails were first acclimated from the baseline temperature of 18 °C to the target temperatures by increments or decreases of 2 °C per hour in a way that all samples reached the target temperature at the same time. The acclimation protocol lasted 12 h. During the warm season, littoral molluscs in the study site (and other intertidal regions of the Persian Gulf) experience daily temperature ranges as large as 10 to 20 °C (Bordbar et al. unpublished data). Since cercarial emergence is likely to be triggered by these gradual temperature changes, we monitored cercarial emergence during the acclimation period. For this, one day before starting the acclimation, snails were individually placed in 50 mL beakers filled with 40 mL of aerated seawater and 2000 µl of a *C. vulgaris* solution (2.5 mg-algae mL^−1^) for a final concentration of 125 µg-algae mL^−1^. During the acclimation, two thermo-bathes (DC10, Thermo Scientific) were used for each target temperature, one as holding tank and the other one set to follow up temperature (2 °C lower or higher). Every 2 h, snails were transferred to the newly set thermo-bath and new beaker filled with 40 mL of seawater pre-equilibrated to the temperature corresponding to the following temperature change and containing dried *C. vulgaris* as a food source (125 µg-algae mL^−1^). The water from the old beaker was placed in a 50 mL falcon tube with 7 mL of 99% ethanol to preserve cercariae. All preserved samples were kept in a fridge at 4 °C, and cercariae were counted at a later stage under a dissecting microscope (Nikon, SMZ1000 body, C-PS160 stand).

After the acclimation phase, the main experiment was conducted over a 3-day period under the same photoperiod described above (start of sunrise at 8:00, maximum white light intensity at 12:00 (50% of the maximum possible intensity), and complete sunset at 20:00. Emerged cercariae were collected at two time points per day: at 8:00 representing the number of released cercariae over the dark period and at 20:00 representing those emerged during the light hours, resulting in a total of 6 cercariae collection points. Sample preservation was performed as described above. During the main experiment we utilize one thermo-bath per temperature.

For both species we reported the minimum emergence temperature threshold (METT), the temperature at which the number of emerging cercariae drops to a level just above zero^86,87^. Previously, this threshold was deemed to be between 20 and 50 cercariae per snail each day. In general, METTs of specific species appear to be 2–3 °C higher than the minimum development temperature threshold (MDTT) where intramolluscan development ceases^86,87^.

### Statistical analysis

All analyses were conducted in R (version 4.1.0) and RStudio© 1.4.1717 (2021R Rstudio, PBC)^88^. The general version of the R scripts can be found in the Supplementary Script.

### Acclimation phase emergence of cercariae

First, we utilized ANOVA and pairwise t-tests and checked that, for each trematode species, the total number of cercariae that emerged during the acclimation phase were not significantly different between samples of various target temperatures (Supplementary Fig. S4).

### Experimental phase emergence of cercariae

Then, for each trematode species, a generalized linear mixed model (GLMM) was developed to explain variation in experimental phase emergence potentially caused by the main and interactive effects of *temperature* (second-order polynomial term), *light regime* (intercept term; two levels: *light* and *dark*), and *time* (second-order polynomial term). In the GLMMs, we also considered the random intercept effect of *snail identity* to account for longitudinal data collection (temporal residual dependence). The GLMMs were fitted using *glmmTMB* function from *glmmTMB* package^85,89^, assuming a quasipoisson residual distribution. Afterward, the model selection was performed using *dredge* function from *MuMIn* package^90^, which applied the Second-order Akaike Information Criterion (AICc) to rank the subsets of the GLMMs (^91^; selected GLMMs are presented in the Supplementary Script). The Wald test was used to evaluate the significance of parameter estimates associated with the selected GLMMs' assumed main and interactive effects. For all GLMMs, assumptions were validated using residual diagnostics tool of the *DHARMa* package^92^ and explained variance (R^2^c) was calculated using *r.squaredGLMM* function from *MuMIn* package^90^. Finally, using *predict* function from *car* package^93^, we predicted the cercarial emergence responses (mean and confidence intervals) to all predictor combinations, which were plotted using *ggplot2*^94^. In addition, the predictions were averaged for each temperature level to plot the overall thermal performance curves and define the optimal temperature for cercarial emergence of each trematode species. Selected model formula for *Acanthotrema tridactyla* and Cyathocotylidae gen. sp. were respectively as followed:

$$\begin{aligned} & glmmTMB\left( {Emergence \, \sim \, Light \, + \, poly\left( {Temperature,2} \right) \, + \, poly\left( {Day,2} \right) \, + \, Light*poly\left( {Temperature,2} \right)} \right. \\ & \quad + \, Light*poly\left( {Day,2} \right) + poly\left( {Day,2} \right)*poly\left( {Temperature,2} \right) \, + \, \left( {1|Sample\_ID} \right), \, family = nbinom1) \\ \end{aligned}$$

$$\begin{aligned} & glmmTMB(Emergence \, \sim \, Light \, + \, poly\left( {Temperature,2} \right) \, + \, poly\left( {Day,2} \right) \, + \, Light*poly\left( {Temperature,2} \right) \, \\ & \quad + \left( {1|Sample\_ID} \right), \, family = nbinom1) \\ \end{aligned}$$

## Supplementary Information

Supplementary Information 1.

Supplementary Information 2.

## Data Availability

All data generated or analysed during this study are included in this published article [and its supplementary information files].
