# Supplementary material for: Thermal optima of cercarial emergence in trematodes from a marine high-temperature ecosystem, the Persian Gulf
Source: Sci Rep. 2023 Mar 25;13:4923. doi: 10.1038/s41598-023-31670-0 (PMC10039888; doi:10.1038/s41598-023-31670-0)
Supplement: Supplementary file 1 — Supplementary Information 1. [file 41598_2023_31670_MOESM1_ESM.docx]

**Supplementary Material** **for “Thermal optima of cercarial emergence in trematodes from a marine high-temperature ecosystem, the Persian Gulf”**

**Content:**

1. **Supplementary Figures and legends**
2. **Supplementary R Scripts**


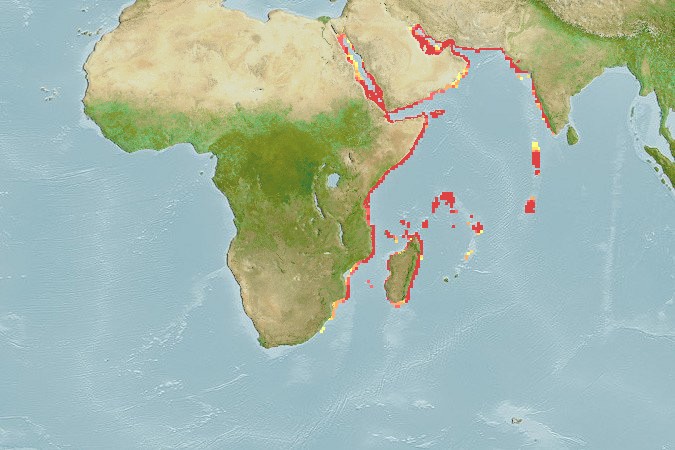

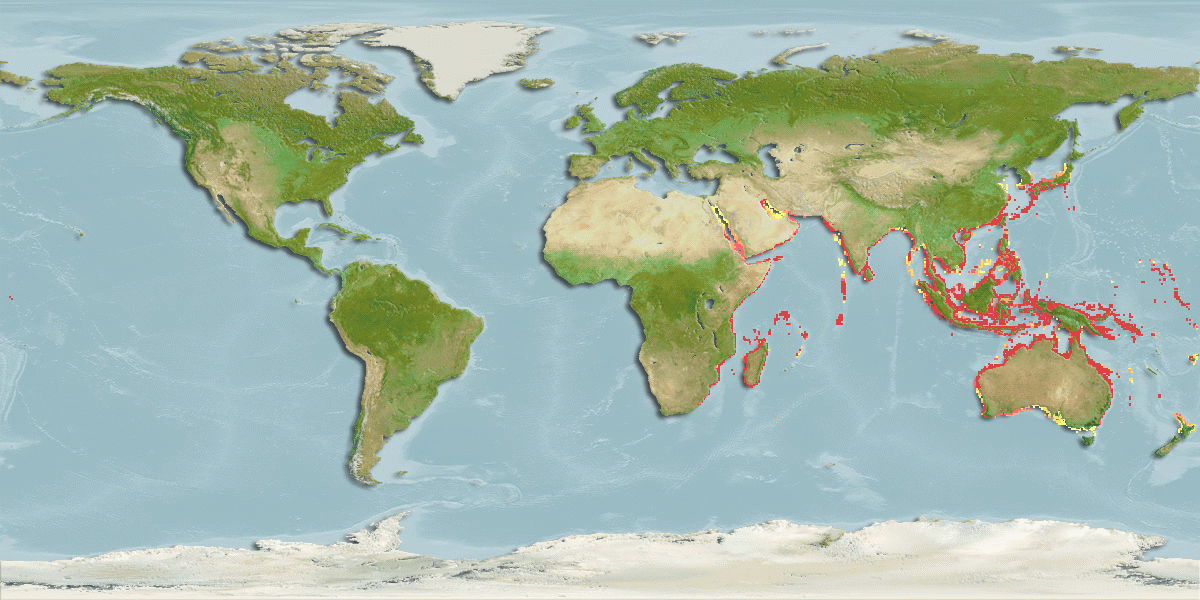


A

B

0.80 – 1.00

0.60 – 0.79

0.40 – 0.59

0.20 – 0.39

0.01 – 0.19

Relative probabilities

of occurrence

**Supplementary Figure S1.** Native habitat of *Pirenella cingulata* (A) and *Aphanius dispar* (B) based on IPCC RCP8.5 emission scenario Retrieved from https://www.aquamaps.org.


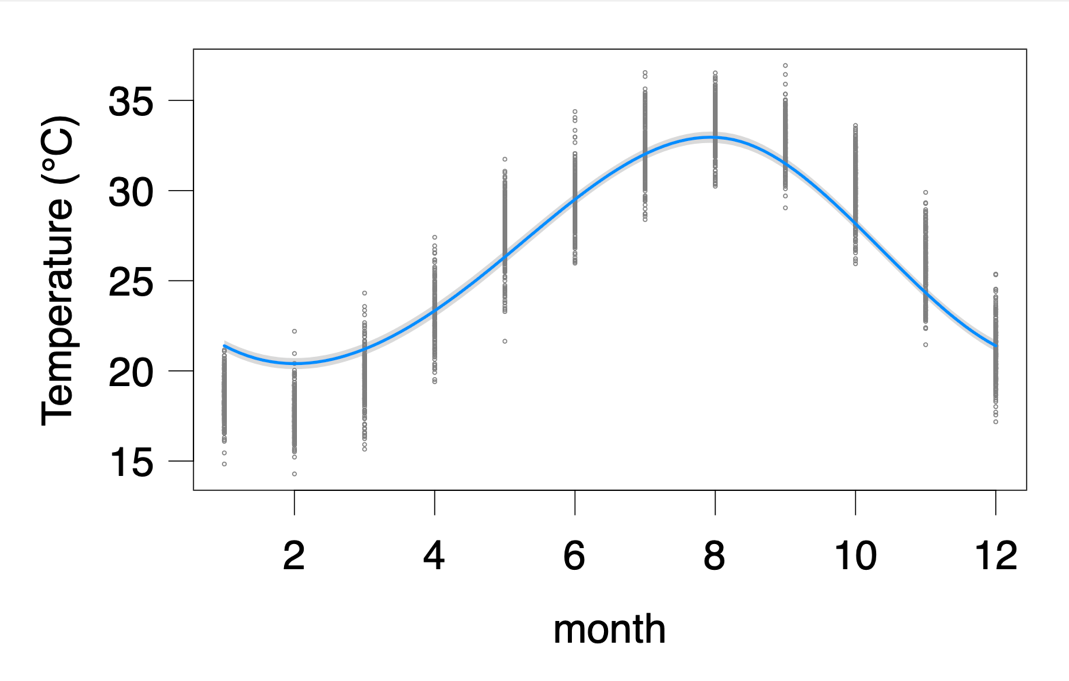
**Supplementary Figure S2.** Averaged monthly Aqua MODIS SST of the sampling locality (Genaveh in the Northern Persian Gulf; 29°33'14.022''N, 50°30'34.0416''E) from 2010-2022 (Data courtesy of NOAA coast watch (<http://coastwatch.pfeg.noaa.gov/erddap)>).


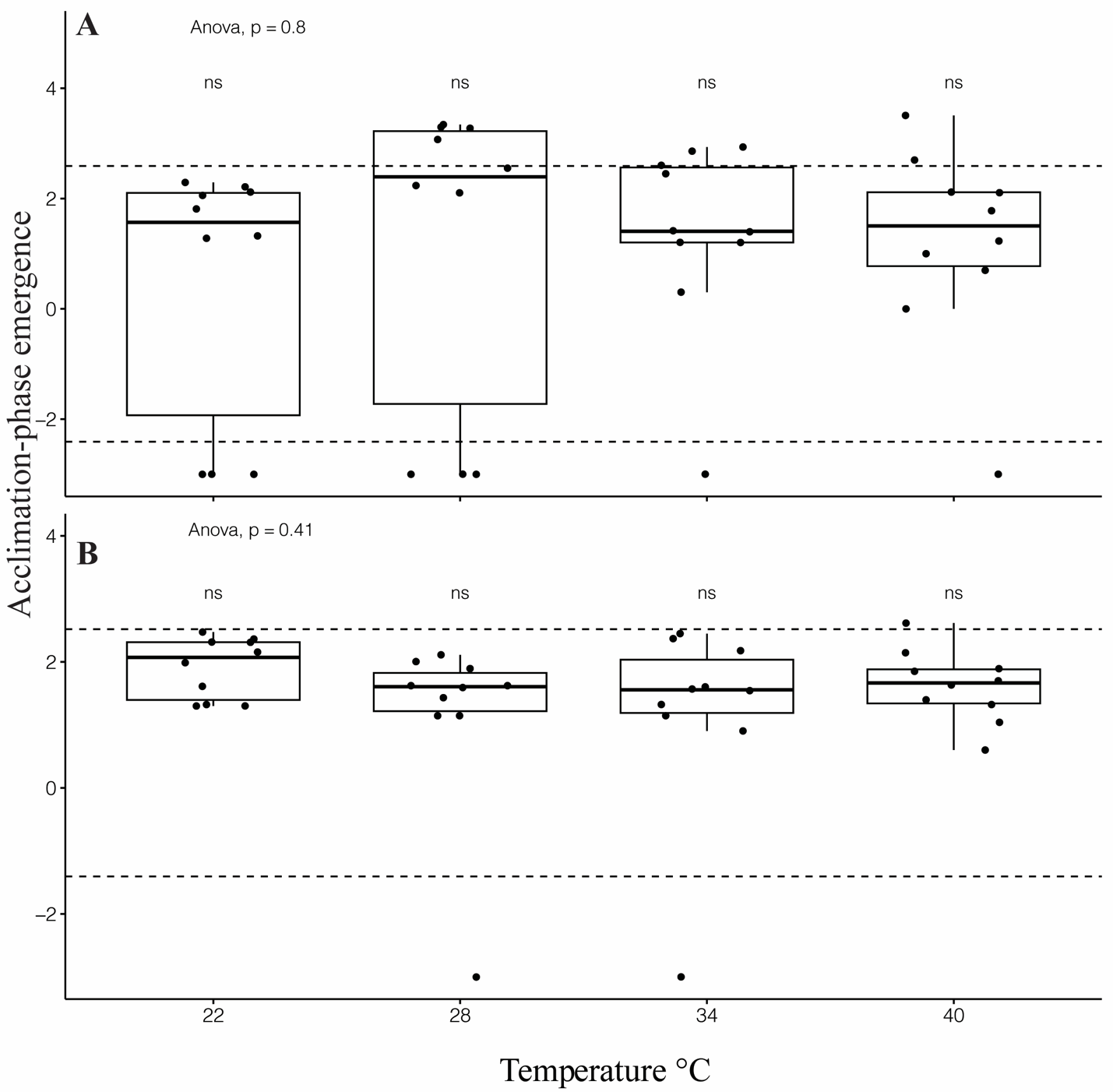
**Supplementary Figure S3.** Box plot compares the total number of the cercarial emergence during the acclimation phase in different temperature for *Acanthotrema tridactyla* (A) and Cyathocotylidae gen. sp. (B). Horizontal dash lines represent standard deviation. **Supplementary Figure S4.** Picture of *Acanthotrema tridactyla* (A) and Cyathocotylidae gen. sp. (B) scale bar 50µm and 100µm, respectively.


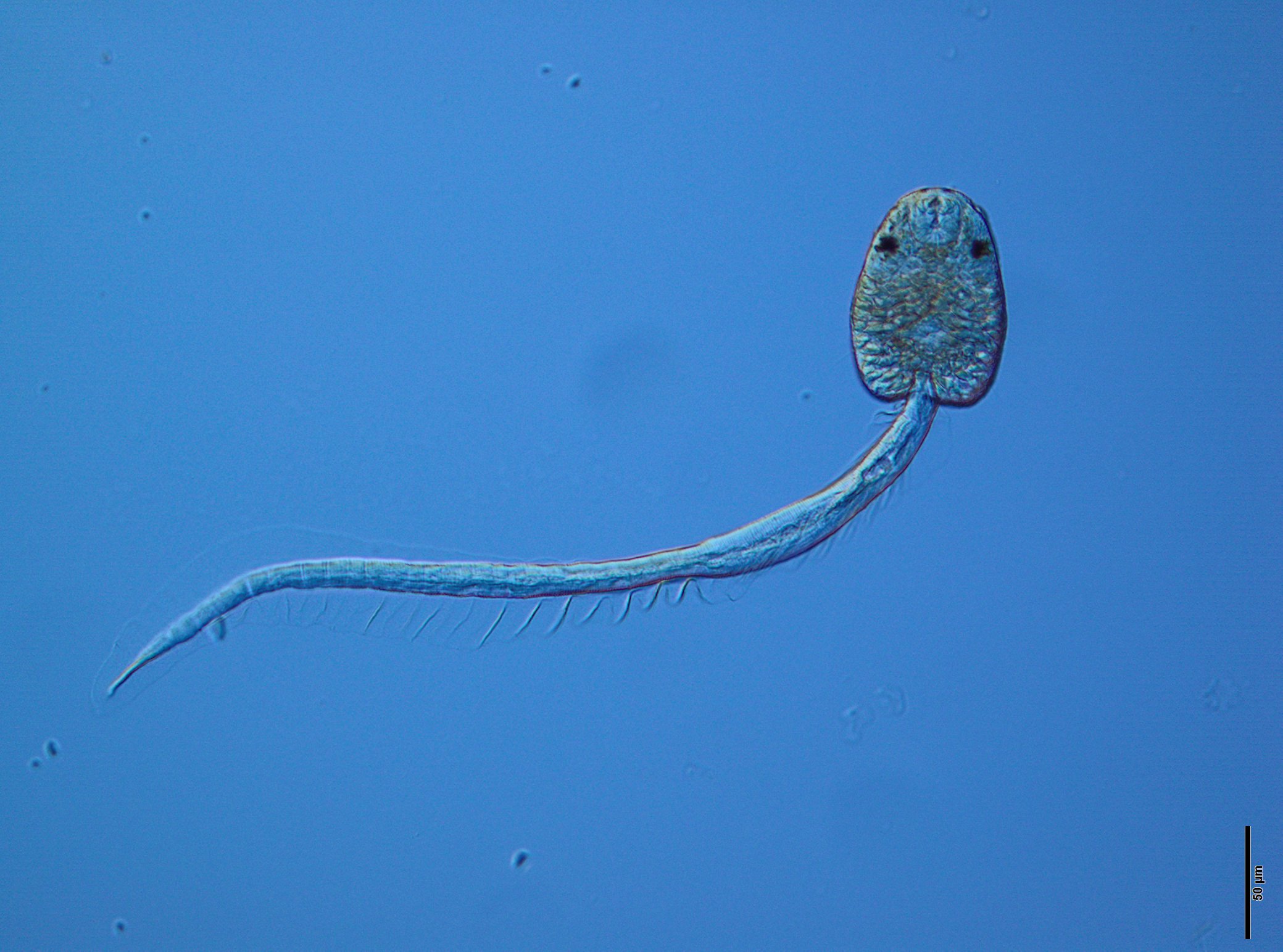

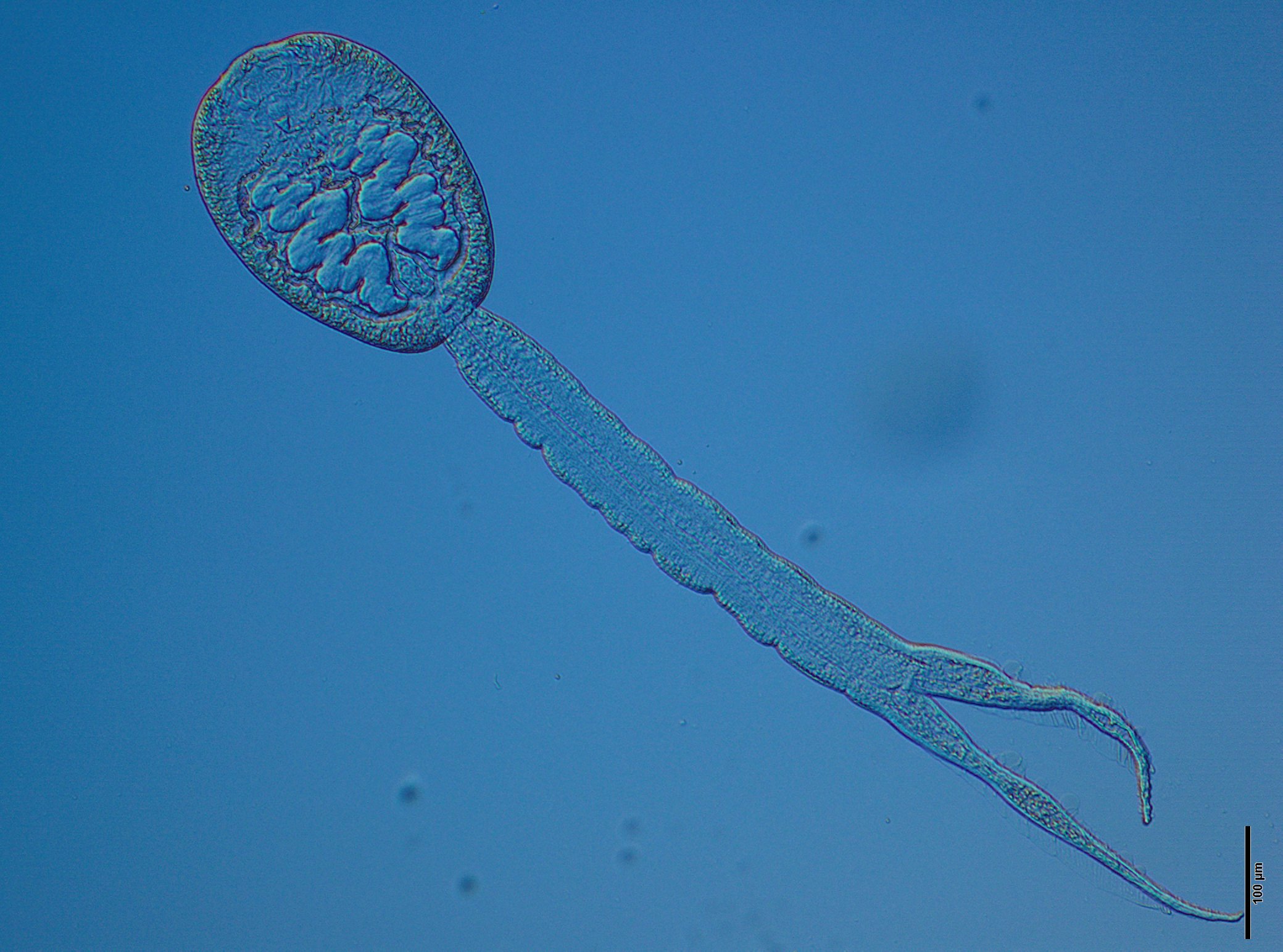


**A**

**B**

**R Scripts**

###Import data frame----

address_main =

address_for_saving = paste()

shedding_total_h = read_excel(paste(address_main, "/shedding_total_h.xlsx", sep=""))

# Inspecting and revising the data frame

df <- shedding_total_h

df <- as.data.frame(df)

head(df)

str(df)

df$Day <- format(df$Date_time, format="%d")

# subset data set separate acclimation from the experiment----

df_accl = subset(df, total_hour <= 12)

df_accl_P = subset(df, total_hour <= 12& Ptype == 'P')

df_accl_F = subset(df, total_hour <= 12& Ptype == 'F')

#sum of emerged cercariae based on Tank during the acclimation----

sum_accl_P = aggregate( shedding ~Sample_ID, data = df_accl_P , FUN = "sum", simplify = TRUE, drop = TRUE)

colnames(sum_accl_P) <- c('Sample_ID','shedding_accl')

sum_accl_F = aggregate( shedding ~Sample_ID, data = df_accl_F , FUN = "sum", simplify = TRUE, drop = TRUE)

colnames(sum_accl_F) <- c('Sample_ID','shedding_accl')

head(sum_accl_F)

df_incu_P = subset(df, total_hour >= 24 & Ptype == 'P' & total_hour <= 84)

df_incu_F = subset(df, total_hour >= 24 & Ptype == 'F' & total_hour <= 84)

tail(df_incu_P)

###

df_incu_P = left_join(df_incu_P, sum_accl_P, by= 'Sample_ID')

df_incu_F = left_join(df_incu_F, sum_accl_F, by= 'Sample_ID')

###----

df_incu_P$Day <- format(df_incu_P$Date_time, format="%d")

number_day_P = aggregate( shedding ~ Day , data = df_incu_P , FUN = "sum", simplify = TRUE, drop = TRUE)

head(df_incu_P)

df_incu_F$Day <- format(df_incu_F$Date_time, format="%d")

number_day_F = aggregate( shedding ~ Day , data = df_incu_F , FUN = "sum", simplify = TRUE, drop = TRUE)

head(df_incu_F)

#Revising variable types

# factors:

df_incu_P$Sample_ID <- as.factor(df_incu_P$Sample_ID)

df_incu_P$Tank<- as.factor(df_incu_P$Tank)

df_incu_P$Light<- as.character(df_incu_P$Time)

df_incu_P$Light<- as.factor(df_incu_P$Light)

df_incu_P$Day<- as.factor(df_incu_P$Day)

df_incu_P$Day<- as.numeric(df_incu_P$Day)

unique(df_incu_P$Light)

##FORCO

df_incu_F$Sample_ID <- as.factor(df_incu_F$Sample_ID)

df_incu_F$Tank<- as.factor(df_incu_F$Tank)

df_incu_F$Light<- as.character(df_incu_F$Time)

df_incu_F$Light<- as.factor(df_incu_F$Light)

df_incu_F$Day<- as.numeric(df_incu_F$Day)

unique(df_incu_F$Light)

str(df_incu_F)

## test correlation of acclimation-phase emergence and exp_emergence for each temperature (3 plots for each species)----

head(df_incu_P)

df_P = aggregate(shedding ~ Sample_ID + shedding_accl + Temperature, data = df_incu_P , FUN = "sum", simplify = TRUE, drop = TRUE)

head(df_P)

dir_file_name = paste(address_for_saving, "cor_accl_incub_para", ".pdf", sep="")

pdf(file = dir_file_name, width = 10, height = 8)

par(mfrow=c(3,2), mar = c(2,3,2,2), cex=0.7,

mgp = c(1, 0.7, 0.1), las=1)

for(i in unique(df_P$Temperature)){

df_P_T = subset(df_P, Temperature == i)

print(i)

plot(df_P_T$shedding ~ df_P_T$shedding_accl, ylab = '', xlab = '')

lines(df_P_T$shedding_accl, predict(lm(df_P_T$shedding ~ df_P_T$shedding_accl)), col = 'grey')

title(main=paste('T =',i,'°C', ' R-squared =', round(cor(df_P_T$shedding, df_P_T$shedding_accl), 2), ' p-value =', round(cor.test(df_P_T$shedding, df_P_T$shedding_accl)$p.value, 2)))

}

dev.off()

ggscatter(subset(df_P, Temperature >=22), x = "shedding_accl", y = "shedding", add = "reg.line",

ylab = 'Experimental emergence', xlab = 'Acclimation-phase emergence') +

stat_cor(aes(label = paste(..rr.label.., ..p.label.., sep = "~`,`~")),

label.x = 3)

ggsave("scatter_para>22.pdf", width = 12, height = 12, units = "cm")

df_P_mean = aggregate(list(df_P$shedding, df_P$shedding_accl), by= list(df_P$Temperature), mean)

colnames(df_P_mean) = c('Temperature', 'shedding', 'shedding_accl')

ggscatter(subset(df_P_mean, Temperature >=22), x = "shedding_accl", y = "shedding", add = "reg.line",

ylab = 'Experimental emergence', xlab = 'Acclimation-phase emergence') +

stat_cor(aes(label = paste(..rr.label.., ..p.label.., sep = "~`,`~")),

label.x = 3)

ggsave("scatter_para_mean>22.pdf", width = 12, height = 12, units = "cm")

head(df_incu_F)

df_F = aggregate(shedding ~ Sample_ID + shedding_accl + Temperature, data = df_incu_F , FUN = "sum", simplify = TRUE, drop = TRUE)

head(df_F)

dir_file_name = paste(address_for_saving, "cor_accl_incub_Forco", ".pdf", sep="")

pdf(file = dir_file_name, width = 10, height = 8)

par(mfrow=c(3,2), mar = c(2,3,2,2), cex=0.7,

mgp = c(1, 0.7, 0.1), las=1)

for(i in unique(df_F$Temperature)){

df_F_T = subset(df_F, Temperature == i)

print(i)

plot(df_F_T$shedding ~ df_F_T$shedding_accl, ylab = '', xlab = '')

lines(df_F_T$shedding_accl, predict(lm(df_F_T$shedding ~ df_F_T$shedding_accl)), col = 'grey')

title(main=paste('T =',i,'°C', ' R-squared =', round(cor(df_F_T$shedding, df_F_T$shedding_accl), 2), ' p-value =', round(cor.test(df_F_T$shedding, df_F_T$shedding_accl)$p.value, 2)))

}

dev.off()

ggscatter(subset(df_F, Temperature >=22), x = "shedding_accl", y = "shedding", add = "reg.line",

ylab = 'Experimental emergence', xlab = 'Acclimation-phase emergence') +

stat_cor(aes(label = paste(..rr.label.., ..p.label.., sep = "~`,`~")),

label.x = 3)

ggsave("scatter_Forco>22.pdf", width = 12, height = 12, units = "cm")

df_F_mean = aggregate(list(df_F$shedding, df_F$shedding_accl), by= list(df_F$Temperature), mean)

colnames(df_F_mean) = c('Temperature', 'shedding', 'shedding_accl')

ggscatter(subset(df_F_mean, Temperature >=22), x = "shedding_accl", y = "shedding", add = "reg.line",

ylab = 'Experimental emergence', xlab = 'Acclimation-phase emergence') +

stat_cor(aes(label = paste(..rr.label.., ..p.label.., sep = "~`,`~")),

label.x = 3)

ggsave("scatter_Forco_mean>22.pdf", width = 12, height = 12, units = "cm")

## box plot of acclimation-phase emergence for each temperature----

accl_df_P = aggregate(shedding_accl ~ Sample_ID + Temperature, data = df_incu_P , FUN = "mean", simplify = TRUE, drop = TRUE)

accl_df_P$log10_shedding_accl = log10(accl_df_P$shedding_accl+0.001)

head(accl_df_P)

accl_df_Pa = subset(accl_df_P, Temperature >=22)

boxplot_P <-ggboxplot(accl_df_Pa, x = "Temperature", y = "log10_shedding_accl",add = "jitter")+#, main = 'Para', ) +

geom_hline(yintercept = mean(accl_df_P$log10_shedding_accl)+ 1*sd(accl_df_P$log10_shedding_accl), linetype = 2) + # Add horizontal line at base

geom_hline(yintercept = mean(accl_df_P$log10_shedding_accl)- 1*sd(accl_df_P$log10_shedding_accl), linetype = 2) + # Add horizontal line at base

stat_compare_means(method = "anova", label.y = 5) + # Add global p-value

stat_compare_means(label = "p.signif",method = "t.test", ref.group = ".all.", label.y = 4)

boxplot_P

ggsave("Box_plot_accl_Para.pdf", width = 24, height = 12, units = "cm")

accl_df_F = aggregate(shedding_accl ~ Sample_ID + Temperature, data = df_incu_F , FUN = "mean", simplify = TRUE, drop = TRUE)

accl_df_F$log10_shedding_accl = log10(accl_df_F$shedding_accl+0.001)

head(accl_df_F)

accl_df_Fo = subset(accl_df_F, Temperature >=22)

boxplot_F <-ggboxplot(accl_df_Fo, x = "Temperature", y = "log10_shedding_accl", add = "jitter") +#main = 'Forco',

geom_hline(yintercept = mean(accl_df_F$log10_shedding_accl)+ 1*sd(accl_df_F$log10_shedding_accl), linetype = 2) + # Add horizontal line at base

geom_hline(yintercept = mean(accl_df_F$log10_shedding_accl)- 1*sd(accl_df_F$log10_shedding_accl), linetype = 2) + # Add horizontal line at base

stat_compare_means(method = "anova", label.y = 4) + # Add global p-value

stat_compare_means(label = "p.signif",method = "t.test", ref.group = ".all.", label.y = 3)

boxplot_F

ggsave("Box_plot_accl_Forco.pdf", width = 24, height = 12, units = "cm")

####PARA_glmmTMB----

# full model

glmNBP_XXX = glmmTMB(shedding ~ Light + poly(Temperature,2) + poly(Day,2) + #poly(shedding_accl,2) +

Light*poly(Temperature,2)*poly(Day,2) + (1|Sample_ID),

family=nbinom1, data = df_incu_P, na.action = "na.fail")

summary(glmNBP_XXX)

pdf("Diagnostic_PARA_Full_glmNBP_XXX.pdf",width=9, height=5)

s=simulateResiduals(fittedModel=glmNBP_XXX,n=250)

s$scaledResiduals

par("mar")

plot(s)

dev.off()

acf(resid(glmNBP_XXX))

pacf(resid(glmNBP_XXX))

?trigamma

MuMIn::r.squaredGLMM(glmNBP_XXX)

pdf("dredge_full_para.pdf")

dr=MuMIn::dredge(glmNBP_XXX)

op <- par(mar=c(2,5,14,3))

plot(dr)

dev.off()

# best model based on AICc (dredge)

glmNBP_XXX = glmmTMB(shedding ~ Light + poly(Temperature,2) + poly(Day,2) +# poly(shedding_accl,2) +

Light*poly(Temperature,2) + Light*poly(Day,2) + poly(Day,2)*poly(Temperature,2) +

(1|Sample_ID), family=nbinom1, data = df_incu_P, na.action = "na.fail")

#AICc(glmNBP_XXX1,glmNBP_XXX)

apatheme=theme_bw(base_size = 11,base_family = "sans")+

theme(panel.grid.major=element_blank(),

panel.grid.minor=element_blank(),

panel.border=element_blank(),

axis.line=element_line())

P<-ggboxplot(df_incu_P, x = "Temperature", y = "shedding",color = "Light")+ facet_wrap("Day")+

scale_color_manual (values = c('#91bfdb','#ef8a62'),

breaks=c("8", "20"),

labels=c("Dark", "Light"), name = "Light regime")+

scale_fill_manual(values = c('#91bfdb','#ef8a62'),

breaks=c("8", "20"),

labels=c("Dark", "Light"),name = "Light regime") +apatheme

P

ggsave(file="Para_inc_box.pdf", width=20, height=10, dpi=1000, units = "cm")

summary(glmNBP_XXX)

sink("Summary_finalm_PARA.txt")

print(summary(glmNBP_XXX))

sink()

pdf("Diagnostic_PARA_FinalM.pdf",width=9, height=5)

s=simulateResiduals(fittedModel=glmNBP_XXX,n=250)

s$scaledResiduals

#graphics.off()

par("mar")

#par(mar=c(1,1,1,1))

plot(s)

dev.off()

MuMIn::r.squaredGLMM(glmNBP_XXX)

### predict and back transform because of log link function of full model----

newdata2 <- expand.grid(Temperature = seq(10,40, by = 0.5),

Light=unique(df_incu_P$Light),

Day=unique(df_incu_P$Day),

#shedding_accl=0,

Sample_ID=unique(df_incu_P$Sample_ID))

preds2 <- predict(glmNBP_XXX, newdata2, se.fit=T, allow.new.levels = F,

re.form=NA, type="link")

newdata2$pred = preds2$fit

newdata2$se = preds2$se.fit

newdata2$ulimit = exp(newdata2$pred + qnorm(0.975)*(newdata2$se))

newdata2$llimit = exp(newdata2$pred - qnorm(0.975)*(newdata2$se))

newdata2$pred = exp(newdata2$pred)

head(newdata2)

newdata2 <- as.data.frame(newdata2)

#take out the prediction for only temperature based on the model predict the max and plot it

df3=aggregate(x = newdata2[c('pred','se','ulimit','llimit')], by=list(newdata2$Temperature), mean)#aggregate based on temperature

df3<- as.data.frame(df3)

head(df3)

### Plotting the full Model PARA----

apatheme=theme_bw(base_size = 11,base_family = "sans")+

theme(panel.grid.major=element_blank(),

panel.grid.minor=element_blank(),

panel.border=element_blank(),

axis.line=element_line(),

axis.title.x=element_blank(),

axis.text.x=element_blank(),

axis.ticks.x=element_blank())

Day.labs = c('20' = "Day 1",'21'= "Day 2",'22'= "Day 3")

#names(Day.labs) =c('20' = "Day 1",'21'= "Day 2",'22'= "Day 3")

ggplot() +

geom_point(data = df_incu_P, aes(x = Temperature,y= shedding, colour=Light),

alpha=1, shape= 16, size=1, position=position_dodge(width=1)) +

geom_line(data= newdata2, aes(x = Temperature, y= pred, colour=Light), size=0.5) +

geom_ribbon(data= newdata2, aes(x = Temperature, y = pred, ymin = ulimit,

ymax =llimit, fill = Light), alpha = .3, colour = NA) +

ylim(-10, 500)+facet_wrap(~Day, labeller = labeller(Day = Day.labs))+

scale_x_continuous(breaks=c(10,16,22,28,34,40))+

scale_color_manual (values = c('#91bfdb','#ef8a62'),

breaks=c("8", "20"),

labels=c("Dark", "Light"), name = "Light regime")+

scale_fill_manual(values = c('#91bfdb','#ef8a62'),

breaks=c("8", "20"),

labels=c("Dark", "Light"),name = "Light regime") +

labs(y = 'Cercarial emergence (no. per snail)')+apatheme

ggsave(file="FinalM_PARA.pdf", width=20, height=7, dpi=1000, units = "cm")

dev.off()

###plot the model for PARA only temperature----

#obtain maximum value from model

apatheme= theme_bw(base_size = 11,base_family = "sans")+

theme(panel.grid.major=element_blank(),

panel.grid.minor=element_blank(),

panel.border=element_blank(),

axis.line=element_line(),

legend.position = "none")

T_opt = df3$Group.1[which.max(df3$pred)]#optimal temperature= 32°C

CE_max = max(df3$pred) # max cercarial release on average =94

plotemerg<-ggplot(df3, aes(x=Group.1, y=pred))+geom_line(color='#E1BE6A') +

geom_ribbon( aes(ymin = llimit, ymax = ulimit,

color=NULL), fill='#E1BE6A' ,alpha=0.25, size=0.5)+

apatheme+ geom_vline(xintercept=T_opt, lty = "dashed", col = "slategray3")+

geom_point(aes(x=T_opt, y= CE_max), colour="red")+

labs(x = 'Temperature (°C)', y = 'Cercarial emergence',

title=NULL,colour="Temperature (°C)")+

scale_x_continuous(breaks=c(10,16,22,28,34,40))

plotemerg

ggsave(file="Emerged cercariae para_temp.pdf", width=16, height=10, dpi=1000, units = "cm")
